# Supplementary material for: Possessing potential weapons (still) heightens anger perception: Replicating and extending a test of error management theory
Source: PLoS One. 2025 Oct 17;20(10):e0326446. doi: 10.1371/journal.pone.0326446 (PMC12533867; doi:10.1371/journal.pone.0326446)
Supplement: S1 Table — Independent samples t-tests comparing emotional and trait ratings between armed and unarmed conditions, disaggregated by rater. Cohen’s d is included as a standardized measure of effect size. * Indicates p <.05. (PDF) [file pone.0326446.s001.pdf]

Table S1. Independent Samples t-tests and Cohen's *d* for Armed vs. Unarmed Conditions

State/Trait	Emotion	Model	t	df	p	Cohen's d
State	Anger	Target	3.640	456.62	<0.001*	0.335
State	Anger	Friend	0.899	470.74	0.369	0.083
State	Disgust	Target	-0.505	470.93	0.614	-0.046
State	Disgust	Friend	0.255	473.95	0.799	0.023
State	Fear	Target	0.679	468.96	0.497	0.062
State	Fear	Friend	0.535	466.86	0.593	0.049
Trait	Anger	Target	2.190	460.32	0.029*	0.202
Trait	Anger	Friend	-0.550	457.70	0.582	-0.051
Trait	Dishonesty	Target	1.546	455.37	0.123	0.143
Trait	Dishonesty	Friend	-1.052	469.00	0.294	-0.097
Trait	Disgust	Target	0.223	459.72	0.824	0.020
Trait	Disgust	Friend	-1.181	473.40	0.238	-0.108
Trait	Fear	Target	0.805	460.75	0.421	0.074
Trait	Fear	Friend	-0.799	467.21	0.425	-0.073
Trait	Unpleasantness	Target	4.438	452.54	<0.001*	0.409
Trait	Unpleasantness	Friend	0.269	465.45	0.788	0.025
Trait	Formidability	Target	-0.586	470.89	0.558	-0.054

Independent samples t-tests comparing emotional and trait ratings between armed and unarmed conditions, disaggregated by rater. Cohen's d is included as a standardized measure of effect size. * Indicates $p < .05$.
