# Supplementary material for: Possessing potential weapons (still) heightens anger perception: Replicating and extending a test of error management theory
Source: PLoS One. 2025 Oct 17;20(10):e0326446. doi: 10.1371/journal.pone.0326446 (PMC12533867; doi:10.1371/journal.pone.0326446)
Supplement: S2 Table — Results from post-hoc Tukey post-hoc HSD test examining the effect of model on state and trait anger. Note significant differences between Model 3 and Models 1 and 2, which do not differ from one another. * Indicates p <.05. (PDF) [file pone.0326446.s002.pdf]

Table S2. Post-Hoc Tukey T-Tests for State and Trait Anger Between Friends

**State/Trait**	**Contrast**	**Diff**	***p***
State	M1-M2	-0.244	0.508
State	M1-M3	0.936	<0.001*
State	M2-M3	1.181	<0.001*
Trait	M1-M2	0.121	0.792
Trait	M1-M3	0.703	<0.001*
Trait	M2-M3	0.582	0.005*

Results from post-hoc Tukey post-hoc HSD test examining the effect of model on state and trait anger. Note significant differences between Model 3 and Models 1 and 2, which do not differ from one another. * Indicates $p < .05$.
