# Supplementary material for: Possessing potential weapons (still) heightens anger perception: Replicating and extending a test of error management theory
Source: PLoS One. 2025 Oct 17;20(10):e0326446. doi: 10.1371/journal.pone.0326446 (PMC12533867; doi:10.1371/journal.pone.0326446)
Supplement: S3 Table — Results from linear regressions predicting state and trait emotional ratings and formidability of the friend based on presence/absence of the target wielding a knife, excluding Model 3. (PDF) [file pone.0326446.s003.pdf]

Table S3. Null Effects of Armed Condition on Perceived State and Trait Emotions of Friend After the Removal of Model 3.

State/Trait	Emotion	*F*	*p*
State	Anger	0.37	0.542
State	Fear	0.10	0.748
State	Disgust	0.04	0.837
Trait	Anger	1.17	0.281
Trait	Fear	1.73	0.188
Trait	Disgust	1.33	0.249
Trait	Dishonesty	3.21	0.074
Trait	Unpleasantness	0.00	0.994
Formidability		0.02	0.898

Results from linear regressions predicting state and trait emotional ratings and formidability of the friend based on presence/absence of the target wielding a knife, excluding Model 3.
