# Supplementary material for: Possessing potential weapons (still) heightens anger perception: Replicating and extending a test of error management theory
Source: PLoS One. 2025 Oct 17;20(10):e0326446. doi: 10.1371/journal.pone.0326446 (PMC12533867; doi:10.1371/journal.pone.0326446)
Supplement: Attachment — Submitted filename: PONE-D-24-25340.pdf [file pone.0326446.s004.pdf]

PLOS ONE

Possessing Potential Weapons (Still) Heightens Anger Perception: Replicating and Extending a Test of Error Management Theory

--Manuscript Draft--

**Manuscript Number:**	PONE-D-24-25340
**Article Type:**	Research Article
**Full Title:**	Possessing Potential Weapons (Still) Heightens Anger Perception: Replicating and Extending a Test of Error Management Theory
**Short Title:**	Possessing Potential Weapons (Still) Heightens Anger Perception
**Corresponding Author:**	Cody Moser<br>University of California Merced<br>Merced, UNITED STATES
**Keywords:**	Error management; Threat perception; Anger; Halo Effect; Social Cognition
**Abstract:**	Error Management Theory (EMT) hypothesizes that humans are functionally biased to err on the side of the least costly mistake when making judgments under uncertainty. Applying EMT to emotion perception, previous studies found that people perceive individuals holding everyday objects with potentially lethal affordances to be both higher in state anger and more anger-prone, relative to individuals holding control objects. Here, we aim to replicate these effects and to exploratorily test whether friends of an individual possessing a potentially lethal tool are also perceived as angrier -- and hence more dangerous -- in a "reverse-halo" effect. Participants (N = 476) were presented an image of an individual depicted as an avid cook and either holding or near a conventional kitchen knife, then asked to rate their emotional states and trait dispositions. Participants were next presented with an image of a second individual framed as a close friend of the cooking enthusiast and asked to assess their emotional states and dispositions. The results replicated prior findings that individuals depicted holding everyday objects with incidentally lethal affordances are perceived as angrier, more prone to anger, and more socially unpleasant. However, we did not find evidence for a "reverse-halo" effect extending to friends. Instead, all of the state and trait emotion ratings of the two individuals were significantly correlated, consistent with an inference (orthogonal to error management motivations) of homophilic similarity in the affective profiles of friends. These results are discussed as they inform prior EMT research and motivate further study of the determinants of emotion perception.
**Order of Authors:**	Cody Moser
	Colin Holbrook
	Richard Ellks
	Ayonna Jones
**Additional Information:**	
**Question**	**Response**
**Financial Disclosure**<br><br>Enter a financial disclosure statement that describes the sources of funding for the work included in this submission. Review the submission guidelines for detailed requirements. View published research articles from *PLOS ONE* for specific examples.<br><br>This statement is required for submission	The author(s) received no specific funding for this work.

and **will appear in the published article** if the submission is accepted. Please make sure it is accurate.<br><br>**Funded studies**<br>Enter a statement with the following details:<br>• Initials of the authors who received each award<br>• Grant numbers awarded to each author<br>• The full name of each funder<br>• URL of each funder website<br>• Did the sponsors or funders play any role in the study design, data collection and analysis, decision to publish, or preparation of the manuscript?<br><br>Did you receive funding for this work?	
**Competing Interests**<br><br>Use the instructions below to enter a competing interest statement for this submission. On behalf of all authors, disclose any competing interests that could be perceived to bias this work—acknowledging all financial support and any other relevant financial or non-financial competing interests.<br><br>This statement is required for submission and **will appear in the published article** if the submission is accepted. Please make sure it is accurate and that any funding sources listed in your Funding Information later in the submission form are also declared in your Financial Disclosure statement.<br><br>View published research articles from *PLOS ONE* for specific examples.	The authors have declared that no competing interests exist.

**NO authors have competing interests**<br><br>Enter: *The authors have declared that no competing interests exist.*<br><br>**Authors with competing interests**<br><br>Enter competing interest details beginning with this statement:<br><br>*I have read the journal's policy and the authors of this manuscript have the following competing interests: [insert competing interests here]*<br><br>* typeset	
**Ethics Statement**<br><br>Enter an ethics statement for this submission. This statement is required if the study involved:<br><br>• Human participants<br>• Human specimens or tissue<br>• Vertebrate animals or cephalopods<br>• Vertebrate embryos or tissues<br>• Field research<br><br>Write "N/A" if the submission does not require an ethics statement.<br><br>General guidance is provided below. Consult the submission guidelines for detailed instructions. **Make sure that all information entered here is included in the Methods section of the manuscript.**	All studies were approved by the University of California, Merced, Institutional Review Board, informed consent was obtained prior to participation, and all methods were in accord with relevant guidelines and regulations.<br><br>For the online experiment, written consent was obtained from participants directly, as is visible in the Study Materials: https://osf.io/jdmt3?view_only=84954626f5954b43bb7af78a2c690ac9

Powered by Editorial Manager® and ProduXion Manager® from Aries Systems Corporation

**Format for specific study types**<br><br>**Human Subject Research (involving human participants and/or tissue)**<br>• Give the name of the institutional review board or ethics committee that approved the study<br>• Include the approval number and/or a statement indicating approval of this research<br>• Indicate the form of consent obtained (written/oral) or the reason that consent was not obtained (e.g. the data were analyzed anonymously)<br><br>**Animal Research (involving vertebrate animals, embryos or tissues)**<br>• Provide the name of the Institutional Animal Care and Use Committee (IACUC) or other relevant ethics board that reviewed the study protocol, and indicate whether they approved this research or granted a formal waiver of ethical approval<br>• Include an approval number if one was obtained<br>• If the study involved *non-human primates*, add *additional details* about animal welfare and steps taken to ameliorate suffering<br>• If anesthesia, euthanasia, or any kind of animal sacrifice is part of the study, include briefly which substances and/or methods were applied<br><br>**Field Research**<br><br>Include the following details if this study involves the collection of plant, animal, or other materials from a natural setting:<br>• Field permit number<br>• Name of the institution or relevant body that granted permission	
**Data Availability**<br><br>Authors are required to make all data underlying the findings described fully available, without restriction, and from the time of publication. PLOS allows rare exceptions to address legal and ethical concerns. See the PLOS Data Policy and FAQ for detailed information.	Yes - all data are fully available without restriction

A Data Availability Statement describing where the data can be found is required at submission. Your answers to this question constitute the Data Availability Statement and **will be published in the article**, if accepted.<br><br>**Important:** Stating 'data available on request from the author' is not sufficient. If your data are only available upon request, select 'No' for the first question and explain your exceptional situation in the text box.<br><br>Do the authors confirm that all data underlying the findings described in their manuscript are fully available without restriction?	
**Describe where the data may be found in full sentences. If you are copying our sample text, replace any instances of XXX with the appropriate details.**<br><br>• If the data are **held or will be held in a public repository**, include URLs, accession numbers or DOIs. If this information will only be available after acceptance, indicate this by ticking the box below. For example: *All XXX files are available from the XXX database (accession number(s) XXX, XXX.).*<br>• If the data are all contained **within the manuscript and/or Supporting Information files**, enter the following: *All relevant data are within the manuscript and its Supporting Information files.*<br>• If neither of these applies but you are able to provide **details of access elsewhere**, with or without limitations, please do so. For example:<br><br>*Data cannot be shared publicly because of [XXX]. Data are available from the XXX Institutional Data Access / Ethics Committee (contact via XXX) for researchers who meet the criteria for access to confidential data.*<br><br>*The data underlying the results presented in the study are available from (include the name of the third party*	All data files including the pre-registration, study materials, analysis script in R, and results from the survey are all archived on OSF at https://osf.io/4c2ue/?view_only=84954626f5954b43bb7af78a2c690ac9.

and contact information or URL).<br>• This text is appropriate if the data are owned by a third party and authors do not have permission to share the data.<br><br>* typeset	
Additional data availability information:	Tick here if the URLs/accession numbers/DOIs will be available only after acceptance of the manuscript for publication so that we can ensure their inclusion before publication.

Latex PDF

Click here to access/download;Manuscript;If_Looks_Could_Kill.pdf

Possessing Potential Weapons (Still) Heightens Anger Perception: Replicating and Extending a Test of Error Management Theory

Cody Moser, Richard Ellks, Ayonna Jones, Colin Holbrook

Department of Cognitive and Information Sciences, University of California Merced, Merced, California, United States

* cmoser2@ucmerced.edu

Abstract

Error Management Theory (EMT) hypothesizes that humans are functionally biased to err on the side of the least costly mistake when making judgments under uncertainty. Applying EMT to emotion perception, previous studies found that people perceive individuals holding everyday objects with potentially lethal affordances to be both higher in state anger and more anger-prone, relative to individuals holding control objects. Here, we aim to replicate these effects and to exploratorily test whether friends of an individual possessing a potentially lethal tool are also perceived as angrier – and hence more dangerous – in a "reverse-halo" effect. Participants ($N = 476$) were presented an image of an individual depicted as an avid cook and either holding or near a conventional kitchen knife, then asked to rate their emotional states and trait dispositions. Participants were next presented with an image of a second individual framed as a close friend of the cooking enthusiast and asked to assess their emotional states and dispositions. The results replicated prior findings that individuals depicted holding everyday objects with incidentally lethal affordances are perceived as angrier, more prone to anger, and more socially unpleasant. However, we did not find evidence for a "reverse-halo" effect extending to friends. Instead, all of the state and trait emotion ratings of the two individuals were significantly correlated, consistent with an inference (orthogonal to error management motivations) of homophilic similarity in the affective profiles of friends. These results are discussed as they inform prior EMT research and motivate further study of the determinants of emotion perception.

Introduction

When faced with incomplete or uncertain information, decision-makers risk two kinds of errors: over-estimations of the information available (leading to false positives, or Type I errors) and under-estimations of the information available (leading to false negatives, or Type II errors). When the costs associated with Type I versus Type II errors are asymmetrical, Error Management Theory (EMT) holds that, under uncertainty, individuals should be biased towards the least costly error (Haselton & Nettle, 2006; Haselton & Galperin, 2012). Within human cognition, EMT encompasses the perception of emotions, which can provide highly fitness-relevant cues of the future behavior of others. Emotion perception is inherently imprecise, in part because individuals are often motivated to mask their own emotional states. When assessing the emotional state of an individual, EMT predicts that their capacity to inflict harm or confer benefits may

be expected to bias emotion-perception toward the least costly error (Galperin et al. 2013). For example, all else being equal, the greater the individual's capacity to inflict harm, the more costly underestimation of their anger becomes (Fessler, Merrill, Holbrook & Ackerman, 2023). Consistent with this model, prior research found that target individuals presented as holding objects with incidentally lethal affordances (i.e., garden shears or a kitchen knife), were estimated as more angry, and more anger-prone, relative to when presented holding contextually equivalent yet non-lethal control objects (i.e., a gardening can or spatula). This effect has replicated both directly (Fessler et al., 2023) and conceptually in meta-analytic examinations (Benjamin Jr et al., 2018).

In addition to perceptions of the emotions of individuals depicted in isolation, a growing literature is beginning to highlight the role that EMT plays in assessing the emotions of individuals depicted in the context of groups (Elias et al., 2017). For example, individuals tend to misclassify crowds as more angry than individuals, particularly in more ambiguous contexts, such as when faces are presented with lower intensities of expression (Mihalache et al., 2021; Gilbert et al., 2011). Insofar as access to allies enhances the perceived formidability of potential antagonists (Fessler & Holbrook, 2013), these effects complement findings that individuals with access to objects which could be leveraged as weapons are perceived to be angrier. Relatedly, previous work on "halo effects" and "horn effects" indicates that positive or negative assessments of individuals embedded within social groups are influenced by assessments of other group members, although these dynamics have yet to be explored with regard to emotion perception (Burns et al., 2021; Remmbers & Martin, 1944).

Here, we sought to integrate the above lines of inquiry by i) replicating prior research into the impact of possession of potentially dangerous tools on perceived anger, while ii) simultaneously exploring whether the hypothesized error management effects on appraisals of target individuals extend to appraisals of their allies. Do participants deem a person as being more angry when they are framed as a friend of someone equipped with a potentially dangerous object? To explore the potential for such a "reverse-halo" effect, we posed parallel questions regarding attributions of state and trait emotions to a target individual and to an individual framed as their friend. In addition, in another exploratory extension of prior work, we collected assessments of the envisioned bodily size and strength of the friend character, as such ratings can index estimates of the danger posed by potential antagonists (Fessler, Holbrook, & Snyder, 2012). To the extent that a target individual's friend is appraised as potentially dangerous, they should be envisioned as both relatively angry and physically formidable.

Materials and methods

Adult U.S. participants were recruited via Prolific.co in exchange for \$0.75. Because we did not know the effect size which may obtain with regard to the anticipated effect of the weapon manipulation on appraisals of the friend character, and because we were replicating elements of Holbrook et al. (2014, Study 1), we simply doubled the sample size used in that study. Per our pre-registration criteria, duplicated participants, smartphone users, individuals with -2.5 SD of duration time in the experiment, and those who failed one of two catch questions were excluded. Following exclusions, the prior study included 264 participants; we therefore recruited 600 participants in the current study during the dates of May 16 - 24, 2022, yielding a final sample of 476 (median age = 34; 64% women, 32% men, 4% other; 78% White).

Closely replicating the between-subjects design used by Holbrook et al. (2014, Study 1), following provision of written informed consent, participants initially viewed a single photo of a man described as enjoying cooking in his everyday life. In the Armed condition, the model was digitally manipulated to be posed holding a kitchen knife. In

the Unarmed condition, the same model was posed empty-handed, with the kitchen knife depicted in a separate window displayed adjacent to the photograph of the model. This presentation held constant the visual features of the stimuli, including potential semantic associations with violence which might be evoked by the image of a knife, while making it clear that the target individual in the control condition was not presently holding the knife. The control model's arm was placed at his side to avoid the impression that their hand was balled in a raised fist when depicted without the grasped object, potentially connoting anger or violent intent (see Figure 1). Participants were asked to rate the model's degree of state anger, fear, and disgust on a scale rating (0 = *Not at all*; 9 = *Extremely*), as well as trait anger, fear, disgust, dishonesty, and unpleasantness (0 = *much less than average*; 9 = *much more than average*). The state and trait questions were presented in separate blocks (randomized), with the order of question items randomized within blocks. The photograph and questions about the target were visible simultaneously.

Next, participants viewed a photo of a neutral male face (taken from the Radboud Faces Database, Langner et al., 2010) described as a close friend of the previous individual and were asked to estimate the same state and trait emotions (see Figure 1). We selected three white male faces to portray the ostensible friend character to hold the apparent race/ethnicity of the target and the friend constant, and thereby avoid potential confounding inferences related to group stereotypes." As before, the state and trait questions were presented in separate blocks (randomized), with the order of items randomized within blocks. The photograph and questions about the target were visible simultaneously. Participants were then asked to estimate the friend's height (in feet and inches)and to rate their size and muscularity using arrays depicting, respectively, silhouettes differing only in size, and bodies differing only in muscularity. Following prior research (e.g., Fessler et al., 2023), these three measures of envisioned bodily formidability (stature, size, and muscularity) were standardized and averaged to create a composite ($a = 0.72$).

Participants were then asked two exploratory questions about their overall degree of confidence in their assessments of both individuals: "Overall, how confident did you feel when rating the feelings and personality traits of the first man you saw [the close friend (the second man you saw)]?" (1 = *Not confident at all*; 9 = *Completely confident*). Finally, participants answered demographic questions and were debriefed.

Pre-registered Hypotheses

We pre-registered several predictions regarding replication of Holbrook et al.'s (2014) findings as well as novel predictions concerning the potential "reverse-halo" or "horn" effect:

As in Holbrook et al. (2014), relative to the target character in the Unarmed condition depicted not holding a knife, we predicted that the target holding a knife would be estimated to be:

1. Higher in state anger
2. Higher in trait anger
3. Lower in trait fear
4. Lower in trait disgust
5. Higher in trait dishonesty
6. Higher in trait unpleasantness

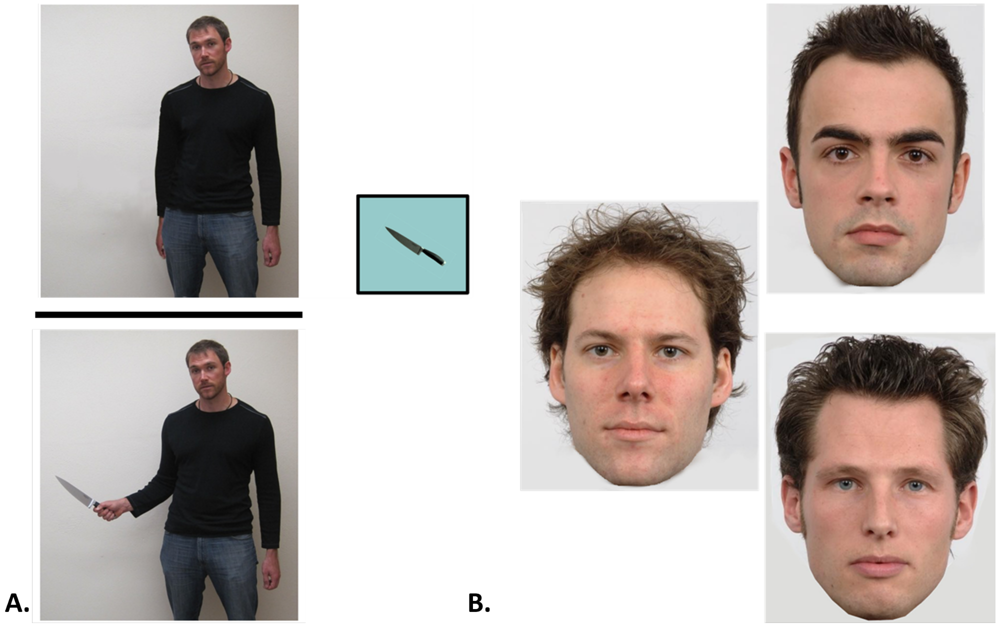

Fig 1. Stimuli representing Target and Friend. Each participant first viewed a man described as enjoying cooking. In the Unarmed condition (Panel A, top) he was depicted adjacent to a kitchen knife; in the Armed condition (Panel A, bottom) he was depicted holding the kitchen knife. Next, participants viewed one of three neutral male faces described as a close friend of the first man (Panel B).

Relative to the friend of the target character in the Unarmed condition depicted not holding a knife, we predicted that the friend of the target holding a knife would be estimated to be:

1. Higher in state anger
2. Higher in trait anger
3. Lower in trait fear
4. Lower in trait disgust
5. Higher in trait dishonesty
6. Higher in trait unpleasantness
7. Higher in envisioned physical formidability

Note that we pre-registered the latter prediction with respect to envisioned physical formidability as contingent on the extent to which the friend character was perceived to be relatively dangerous (operationalized here according to how angry they were appraised to be), anticipating that potential effects on bodily estimates may be quite modest given the relatively weak and indirect connection to danger (i.e., alliance with a friend who happens to have access to a conventional kitchen knife). Indeed, the relatively weak connection to danger cues inherent to this manipulation (e.g., framing the character as a close friend of a cook rather than a violent criminal) renders the predictions with regard to state and trait emotion as well as envisioned physical formidability highly conservative tests of the impact of EMT considerations to social appraisals.

All statistical analyses were conducted in R (Team et al., 2013). We employed a general linear model, controlling for which friend face model was displayed, to assess the effects of weapon manipulation on state and trait ratings of the target and the friend. To assess the possibility that, for reasons orthogonal to EMT, participants would infer homophily between the target and his friend, we similarly ran correlations of the state and trait ratings between the two individuals (Laakasuo et al. 2017; Selfhout et al., 2010). All preregistrations, data, scripts, and materials are available at `https://osf.io/4c2ue/`

Results

Replication of Holbrook et al. (2014)

We observed significant differences in both state (mean for knife condition: 4.36 ,SD: 2.31; no knife: 3.64, SD: 2.0) and trait anger (mean for knife condition: 5.36, SD: 1.79; no knife: 5.02, SD: 1.61) between the armed and unarmed conditions, indicating support for these predictions (see Table 1). Against our other predictions, we found no differences for trait fear, disgust, or dishonesty (see Figure 2 to visualize rating distributions). In support of our final prediction, the Armed target was rated more trait unpleasant than the Unarmed target (mean for knife condition: 4.98, SD: 1.76, no knife: 4.31, SD: 1.49).

With regards to the friend state and trait anger and trait unpleasantness, there were strong effects for the model. We conducted post-hoc Tukey comparisons, finding that Model 3 was significantly higher in state and trait anger than both Models 2 and 3, while the two themselves did not differ (SI Table 1). After analyzing participants which only received Models 1 or 2, our results remained robust, with the exception of a weaker effect for Trait Anger (p = 0.054) (SI Tables 2 & 3).

Table 1. Effects of Armed Condition on Perceived State and Trait Emotions.

State/Trait	Emotion	F	p
State	Anger	13.51	<0.001*
Trait	Anger	5.65	0.018*
Trait	Fear	0.72	0.396
Trait	Disgust	0.05	0.820
Trait	Dishonesty	2.92	0.088
Trait	Unpleasantness	20.3	<0.001*

Results from linear regressions predicting state and trait emotional ratings of the target based on possession versus non-possession of a knife. * Indicates p-value less than 0.05.

Tests of a potential "reverse halo" effect

We ran multiple linear regressions on friend state and trait anger with both friend state and trait anger and condition as our predictor variables. Against our predictions for the friend, we found no effects of condition on ratings of the friend character (see Table 2). In line with our exploratory test of the possibility that participants would infer affective homophily between friends, we observed significant correlations between all of the state and trait assessments of both characters.

Figure 2 shows the results from both the state and trait ratings of both the target and friend, by condition. As can be seen most, means are similar for each variable, with the exception of state anger and traits anger and unpleasantness for the target.

Table 2. Null Effects of Armed Condition on Perceived State and Trait Emotions of Friend.

State/Trait	Emotion	*F*	*p*
State	Anger	0.70	0.402
Trait	Anger	0.25	0.619
Trait	Fear	0.40	0.528
Trait	Disgust	1.08	0.299
Trait	Dishonesty	1.06	0.304
Trait	Unpleasantness	0.11	0.738

Results from linear regressions predicting state and trait emotional ratings and formidability of the friend based on presence/absence of the target wielding a knife.

Tests of Inferred Homophily between Target and Friend

With regards to inferences of affective homophily, we found highly significant linear positive correlations (rs 0.29-0.48, ps $<.001$) between the two characters for all state and trait measures (Table 3).

Table 3. Correlations Between Target and Friend State and Trait Ratings.

State/Trait	Emotion	β	*p*
State	Anger	0.33	<0.001
State	Disgust	0.36	<0.001
State	Fear	0.29	<0.001
Trait	Anger	0.34	<0.001
Trait	Disgust	0.39	<0.001
Trait	Fear	0.32	<0.001
Trait	Dishonesty	0.48	<0.001
Trait	Unpleasantness	0.40	<0.001

Results from linear regressions predicting friend state and trait emotional ratings from the same state and trait emotional ratings of the target as tests of homophily; e.g. in the case of State Anger, friend state anger is predicted based on the state anger of the target.

Discussion

We found that possession of an everyday object with incidentally lethal affordances heightened perception of state and trait anger, as well as trait unpleasantness, consistent with EMT and replicating previously observed effects (Holbrook et al., 2014; also see Fessler et al., 2023). However, departing from prior findings, we observed no effects of the object manipulation on trait fear, disgust, or dishonesty, potentially owing to the greater power in this study (N=476) compared to the initial study (N=264). The overall pattern of results nonetheless supports the prediction that dangerous object possession up-regulates perception of anger and related traits in a manner consistent with an adaptive capacity to minimize risk of potentially costly aggression.

Extending prior research, we tested whether heightening perceived anger due to framing an individual as possessing a potentially lethal object would similarly heighten perceptions of the anger or physical formidability of a friend. We found, contrary to

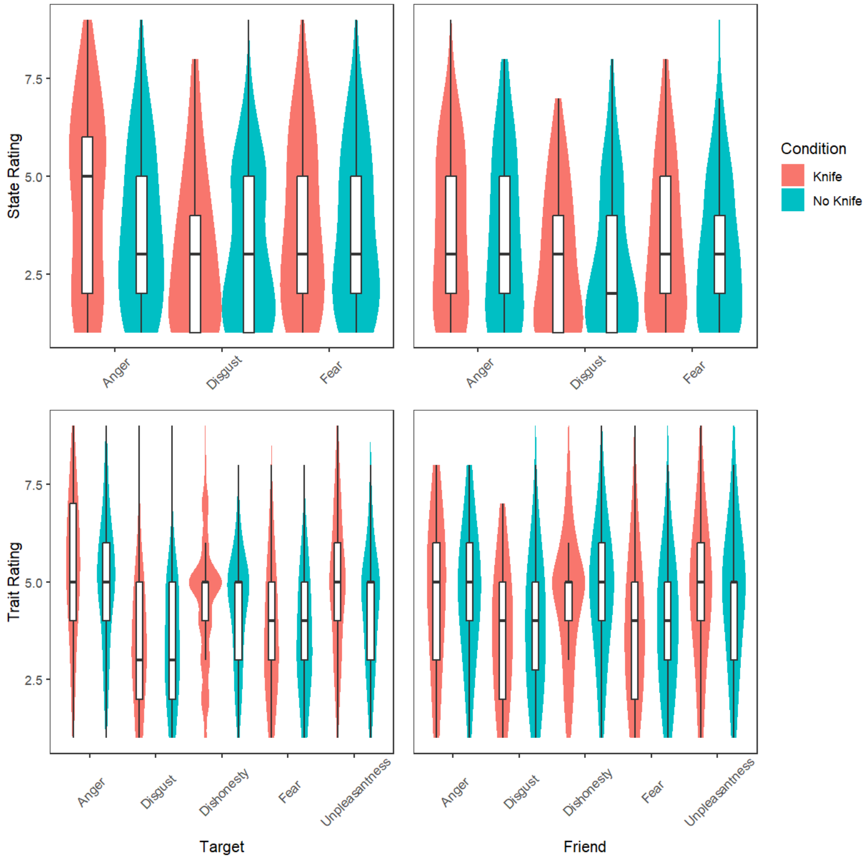

Fig 2. State and Trait Ratings for Target and Friend Top Row: Ratings by participants of emotional states of both the target individual (left) and his friend (right) dependent on whether the friend was presented holding a knife or without a knife. Bottom Row: Ratings by participants of emotional traits of target individual (left) and his friend (right) dependent on the same condition as above.

predictions, that the object manipulation had no effect on any of the appraisals of the friend. While this null finding may be interpreted as evidence against the proposed "reverse-halo" effect with respect to appraisals of allies, two methodological limitations should be noted. First, whereas prior research examining the emotional assessment of members of groups have presented individuals together, our experiment presented the two individuals sequentially, potentially minimizing conceptual associations between the target person and their friend, including the specific impression that should the armed individual attack, their friend would be local enough to be able to join them. Second, the online approach employed here inherently lacks ecological validity. Indeed, given that the individual holding a knife is depicted using a mere photograph on a computer screen–and hence not likely to pose any real-world danger to the participant–it is remarkable that observers reliably perceive this person as relatively angry and unpleasant in character. Were a person possessing a potentially lethal object and accompanied by an ostensible friend encountered in a lab space (e.g., using research confederates), or encountered as characters in a realistic simulation (e.g., using augmented or virtual reality), friends might indeed be perceived as relatively angry, unpleasant, and formidable along with to the person holding the object. Thus, although the present study does not provide support for a "reverse-halo" effect on anger

perception, future work employing more valid methods is necessary to rule out this hypothesis.

Finally, in an exploratory test of the extent to which friends would be inferred to possess similar emotional profiles, we examined whether the target's trait and state emotions correlated with those of the friend. Indeed, consistent with inferences of affective homophily and prior research (Laakasuo et al. 2017; Selfhout et al., 2010), and despite the lack of an effect of manipulation on ratings of the friend, we observed strong correlations between all of the state and trait ratings. These positive results motivate future work on the role of inferred homophily in emotion perception and detection.

In closing, we successfully replicated previously documented shifts in attributions of state anger and related antisocial traits (i.e., anger-proneness, social unpleasantness) caused by manipulation of a highly subtle contextual determinant: the target individual's possession of an ordinary kitchen knife in their hand versus ownership of the same knife in an unspecified location. Although we have focused here on effects of dangerous object possession on perception of threat-relevant emotions and traits, EMT predicts analogous effects of subtle context cues on functional biases in emotions and traits relevant to the potential conferral of benefits (e.g., romantic interest, career opportunities). We encourage affective scientists to draw upon EMT when generating hypotheses regarding contextual determinants of emotion categorization.

Acknowledgments

We thank Alejandro Pérez Velilla for feedback on the study during its initial design phase.

References

1. Haselton, M. G., & Nettle, D. The Paranoid Optimist: An Integrative Evolutionary Model of Cognitive Biases. Personality and Social Psychology Review, 10(1), 47-66. 2006. https://doi.org/10.1207/s15327957pspr1001_3

2. Haselton M, Galperin A. Error management and the evolution of cognitive bias. Interpers. Behav. 45. 2012;63.

3. Galperin A, Fessler DM, Johnson K, Haselton M. Seeing Storms Behind the Clouds: Biases in the Attribution of Anger. Evolution and Human Behavior. 2013 Sep 1;34(5):358-65.

4. Fessler DM, Merrell W, Holbrook C, Ackerman J. Beware the foe who feels no pain: Associations between relative formidability and pain sensitivity in three US online studies. Evolution and Human Behavior. 2023 Jan 1;44(1):1-0.

5. Fessler, D. M., Merrell, W., Holbrook, C., & Ackerman, J. Beware the foe who feels no pain: Associations between relative formidability and pain sensitivity in three US online studies. Evolution and Human Behavior. 2023 Jan 1;44(1):1-0.

6. Benjamin Jr AJ, Kepes S, Bushman BJ. Effects of weapons on aggressive thoughts, angry feelings, hostile appraisals, and aggressive behavior: A meta-analytic review of the weapons effect literature. Personality and Social Psychology Review. 2018 Nov;22(4):347-77.

7. Elias, E., Dyer, M., & Sweeny, T. D. Ensemble Perception of Dynamic Emotional Groups. Psychological science. 2017 Feb;28(2):193-203.

8. Mihalache, D., Lamer, S. A., Allen, J., Maher, M., & Sweeny, T. D. Anger bias in the evaluation of crowds. Journal of Experimental Psychology: General. 2021 Sep;150(9):1870.

9. Gilbert, T., Martin, R., & Coulson, M. Attentional biases using the body in the crowd task: are angry body postures detected more rapidly?. Cognition and Emotion. 2011 Jun 1;25(4):700-8.

10. Fessler, D.M.T., & Holbrook, C. Friends shrink foes: The presence of comrades decreases the envisioned physical formidability of an opponent. Psychological science. 2013 May;24(5):797-802.

11. Burns, E. J., Yang, W., & Ying, H. Friend effects framework: Contrastive and hierarchical processing in cheerleader effects. Cognition. 2021 Jul 1;212:104715.

12. Remmers HH, Martin RD. Halo effect in reverse—are teachers' ratings of high-school pupils valid?. Journal of Educational Psychology. 1944 Apr;35(4):193.

13. Fessler DM, Holbrook C, Snyder JK. Weapons make the man (larger): Formidability is represented as size and strength in humans. PloS one. 2012 Apr 11;7(4):e32751.

14. Holbrook C, Galperin A, Fessler DM, Johnson KL, Bryant GA, Haselton MG. If looks could kill: Anger attributions are intensified by affordances for doing harm. Emotion. 2014 Jun;14(3):455.

15. Langner O, Dotsch R, Bijlstra G, Wigboldus DH, Hawk ST, Van Knippenberg AD. Presentation and validation of the Radboud Faces Database. Cognition and emotion. 2010 Dec 1;24(8):1377-88.

16. RTeam. Core. R: A language and environment for statistical computing. R Foundation for Statistical Computing, Vienna, Austria. Online: http://www.R-project.org. 2013;201.

17. Laakasuo, M., Rotkirch, A., Berg, V., & Jokela, M. The company you keep: Personality and friendship characteristics. Social Psychological and Personality Science. 2017 Jan;8(1):66-73.

18. Selfhout, M., Burk, W., Branje, S., Denissen, J., Van Aken, M., & Meeus, W. Emerging late adolescent friendship networks and Big Five personality traits: A social network approach. Journal of personality. 2010 Apr;78(2):509-38.

Supplementary Materials

Table 4. Post-Hoc Tukey T-Tests for State and Trait Anger Between Friends

State/Trait	Contrast	Diff	*p*
State	M1-M2	-0.244	0.508
State	M1-M3	0.936	<0.001*
State	M2-M3	1.181	<0.001*
Trait	M1-M2	0.121	0.792
Trait	M1-M3	0.703	<0.001*
Trait	M2-M3	0.582	0.005*

Results from post-hoc Tukey post-hoc HSD test examining the effect of model on state and trait anger. Note significant differences between Model 3 and Models 1 and 2, which do not differ from one another. * Indicates p-value less than 0.05.

Table 5. Effects of Armed Condition on Perceived State and Trait Emotions After the Removal of Model 3.

State/Trait	Emotion	*F*	*p*
State	Anger	9.26	<0.003*
Trait	Anger	3.74	0.054
Trait	Fear	0.20	0.657
Trait	Disgust	0.14	0.710
Trait	Dishonesty	0.36	0.551
Trait	Unpleasantness	17.0	<0.001*

Results from linear regressions predicting state and trait emotional ratings of the target based on possession versus non-possession of a knife, excluding Model 3. * Indicates p-value less than 0.05.

Table 6. Null Effects of Armed Condition on Perceived State and Trait Emotions of Friend After the Removal of Model 3.

State/Trait	Emotion	*F*	*p*
State	Anger	0.49	0.482
Trait	Anger	1.24	0.266
Trait	Fear	2.05	0.153
Trait	Disgust	1.53	0.217
Trait	Dishonesty	3.55	0.061
Trait	Unpleasantness	0.00	0.955

Results from linear regressions predicting state and trait emotional ratings and formidability of the friend based on presence/absence of the target wielding a knife, excluding Model 3.
