# Supplementary material for: Possessing potential weapons (still) heightens anger perception: Replicating and extending a test of error management theory
Source: PLoS One. 2025 Oct 17;20(10):e0326446. doi: 10.1371/journal.pone.0326446 (PMC12533867; doi:10.1371/journal.pone.0326446)
Supplement: Attachment — Submitted filename: PLOS Response.pdf [file pone.0326446.s005.pdf]

Replies to Editor

As the recommendations appear to have been generated with the use of a Large Language Model, many of the suggestions lacked contextually relevant understanding of the hypotheses, literature or scholarly norms. In our revision, accordingly, we have primarily focused on addressing the feedback generated by the reviewers. However, we also carefully considered the AI-generated feedback, and did find some substantive suggestions which we address below.

- **Suggestion of an alternative title**

  One suggestion was that the current title may be too jargon-laden. The alternative titles suggested, were either so broad as to not convey the key findings of the study (e.g. “"The Role of Potential Weapons in Heightening Anger Perception: A Test of Error Management Theory"), or were actually lengthier and more jargony than the current title (e.g. “"How Potential Weapons Influence Anger Perception: A Replication and Expansion of Error Management Theory"). We concluded that the current title, which straightforwardly states the findings of the current study and straightforwardly identifies it as a replication, is more appropriate.

- **Suggestion of a simpler abstract**

  Another line of feedback was that the abstract, as it is written, does not emphasize the study’s novelty and that we overemphasize the null findings. PLOS-ONE’s mission statement as a journal prioritizes replicable, open science over novelty or asymmetrically highlighting significant effects over nulls. Indeed, we identified PLOS as a suitable venue for this project in part because the key significant findings replicate previously published work. While not novel, this sort of work is indispensable for cumulative science to proceed. Therefore, we do not believe that it would be appropriate or necessary to minimize our null results or reframe the results in a way that would attempt to exaggerate novelty. It was also suggested that details of our participant sample be provided directly in the abstract, whereas scholarly conventions dictate that such details are left to readers in the text.

- **Introduction of terminology in Introduction Section**

  In the introduction section, the AI argues that we have overused jargon and not clearly defined our key terms, such as Error Management Theory and Type I and Type II errors. To the contrary, these terms are defined in the first two sentences (lls. 2-6) before they are employed in the text.

  > “When faced with incomplete or uncertain information, decision-makers risk two kinds of errors: over-estimations of the information available (leading to false positives, or Type I errors) and under-estimations of the information available (leading to false negatives, or Type II errors). When the costs associated with Type I versus Type II errors

are asymmetrical, Error Management Theory (EMT) holds that, under uncertainty, individuals should be biased towards the least costly error (Haselton & Nettle, 2006; Haselton & Galperin, 2012)."

- **Methods**

The AI stated that we should clarify our sample size and exclusion criteria, which were clarified in lls. 50-55 of the text.

> "Because we did not know the effect size which may obtain with regard to the anticipated effect of the weapon manipulation on appraisals of the friend character, and because we were replicating elements of Holbrook et al. (2014, Study 1), we simply doubled the sample size used in that study. Per our pre-registration criteria, duplicated participants, smartphone users, individuals with -2.5 SD of duration time in the experiment, and those who failed one of two catch questions were excluded."

Additionally, bias in face selection (that we selected three white males faces) was brought up as a concern, which we addressed in lls. 91-93 of the text.

> "We selected three white male faces to portray the ostensible friend character to hold the apparent race/ethnicity of the target and the friend constant, and thereby avoid potential confounding inferences related to group stereotypes."

- **Results**

The primary concern regarding our results was the effect of Model 3. In the manuscript (lls. 153-158), this is discussed at length, and we analyze our results with and without these Models in SI Tables 1, 2, and 3. Similarly, while the feedback states we did not describe what is controlled for in our linear model, this is explicitly stated in lls. 141-143 of the text.

- **Discussion**

Finally, the feedback notes 1) cognitive demands and 2) a lack of ecological validity in our approach. These issues are acknowledged and explored in a lengthy paragraph (lls. 183-205).

- **Reference Issues**

As reference 2 was incomplete, we have updated our reference list. Additionally, as references 4 and 5 were duplicated, we have removed one.

> Haselton M. G., Galperin A. Error management and the evolution of cognitive bias. In: Social Thinking and Interpersonal Behavior. Psychology Press; 2013. p. 45-63

Replies to Reviewer 1

R1.1: “Where in USA was this research carried out? This is not stated. A brief description of the study site (population and ethnic composition included) will assist us appreciate the study conditions and also agree/or not on the appropriateness of the study sample.”

We thank Reviewer 1 for their comment on our paper. In the original version of the manuscript, we state on line 49 that participants were recruited online via Prolific. As we did not want to violate privacy concerns in our dataset and believed our sample size was large enough to capture a wide enough distribution of participants across the United States, we did not collect location data from participants in this study. Nevertheless, we believe our online sample size likely captures a wide variety of Americans who participate in studies online.

To clarify our recruitment for the reasons given by the Reviewer, we have added additional text (ll. 49) to the Materials and methods stating that adult participants were recruited from throughout the United States.

> “Adult participants were recruited from throughout the United States via Prolific.co in exchange for $0.75.”

Replies to Reviewer 2

R2.1: “This manuscript professes to replicate previous findings about Error Management Theory (EMT) and to extend the findings to examine potential “halo-effects” to neutral associated individuals. In sum, I find the research design and empirical evaluations to be acceptable (with a possible exception), and the results sound given the experiment.”

We thank Reviewer 2 for their work in reviewing our paper and generous appraisal of our approach.

R2.2: “First and foremost, this is not my area of expertise. While I am competent to evaluate the research design and implementation, this literature is foreign to me, and I do not mean to suggest otherwise in my review. With this caveat noted, here are my impressions. First, I am a bit surprised to see the results of this experiment being expressed in terms of linear regression models. Given the authors’ use of Latex and R, I would have expected a more sophisticated modeling schema, especially given the truncated data and non-linear comparisons. Conversely, dealing with experimental design in this manner, I would have expected to see the discussion of and the results presented of a Latin Square design. Not that this matters much, as the OLS models will replicate the more conventional ANOVA presentation – it's just interesting.”

We believe that the Reviewer’s interpretation of the analysis we conducted in our study may be due to a difference between disciplines. We agree with the Reviewer that this question could have been approached from a range of different statistical models - in particular, using a signal detection approach with binary logistic regression. However, we chose to utilize conventional ANOVA and linear regression models to facilitate comparison to the results of the original Holbrook et al. study. Nevertheless, we agree with the author that other models might have been used and would have conceptually replicated our results.

R2.3: “I see no deficits in the empirical modeling nor in the interpretation of that modeling. The authors do an excellent job of qualifying the findings. Similarly, I have no problems with the sampling strategy or sample size. My concerns with this manuscript are twofold. First, the treatment illustration portrays an individual with a chef’s knife in a partially extended arm. My concern here is that attitudinal data of perceived threats extends beyond inanimate objects to body position and posturing. Given that this is a somewhat atypical posture for a “cooking enthusiast” to hold a knife, I have concerns about the validity of the neutral armed image.”

The Reviewer raises the ecological concern that our treatment condition of the target holding a knife may be more threatening in appearance based on the target’s stance rather than on the object being held. While we agree this could pose a confound for the experiment, in the sense that the target may be primed, we note that in the original study, the model was presented with several additional objects in the same stance (as these objects were photoshopped in): a spatula, garden shears, and a flowercan. In this case, only the image with the knife or the garden shears (both objects with lethal affordances) were seen as threatening by participants. In addition, the framing of the photograph to participants explained that the models had been asked to pose for a photo while holding an object illustrating their hobby, rather than while performing their hobby (i.e., cooking). As such, it would make sense for the model to hold and display the object in this way for the photo. We thus believe that the condition with the knife in our study likely replicates the original findings while serving as an ideal priming mechanism for our extension examining the influence (or, in our case, lack thereof) that the target has on ratings of a friend. In the revision, we have added an acknowledgment of this issue as a potential limitation, reminded readers of the framing explaining why a cooking enthusiast would hold the knife in this way for the photograph, and described how this potential confound was handled in the original paper (i.e., by utilizing alternate objects held with arm extended) (lls. 62-73).

> "The model is depicted holding the knife out to his side, which might be interpreted as having been perceived as implicitly threatening, and thereby confounding the lethal affordance of this every day item with an aggressive motive in the model. We do not believe that this interpretation plausibly explains our findings, for two reasons. First, participants were informed that the models had been asked to pose for a photo while holding an object intended to illustrate their hobby, hence the model's stance should not have conveyed threatening intent, but rather cooperatively presenting their hobby-relevant object to the camera, as asked. Second, in the original study, the same model was compared while holding different objects (a spatula, garden shears, and a flowercan) in the exact same stance, and the objects lacking lethal affordances elicited significantly distinct effects on state or trait emotion attribution relative to the knife or shears."

R2.4: "Secondly, and far less important, I am skeptical that this replication presents much in the way of substantive contribution to the literature – but that is a question far better suited for someone with better knowledge of the literature."

We disagree that our study does not make a contribution to the literature for two reasons. First, in terms of the replication, the original Holbrook study, at 10 years old, has been due for a replication given recent failures in the priming literature, a method which is suggested by the methodology of this study. Perhaps more to the point, novelty should not be considered a more valuable aspect of research published in PLOS than replicability, as one of the reasons the journal was created was to encourage replication projects in service of cumulative, reliable science.

Replies to Reviewer 3

R3.1: The target manuscript reports the results of one study. Its primary goal is to replicate the results of Halbrook et al (2014, study 1), with a secondary goal of exploring the so called "reverse-halo effect". The manuscript is very well written (I found only a typo in page 3, line 82: the sentence ends with quotation marks), clear and rigorous while also managing to be engaging – it was truly a pleasure for me to read this work. I also congratulate the authors for their effort to replicate an important result – despite its widely acknowledged relevance, it is unfortunate that most authors seldomly devote their efforts to replications.

We thank the reviewer for their generous sentiments regarding our study and current version of the manuscript, as well as for their close reading of the text. We have updated the error the author found on page 3 and removed the quotation marks. We agree that replication is invaluable!

R3.2: “In regards to the secondary goal – exploring the "reverse-halo effect" – there is room for improvement and I could easily suggest some ways to strengthen the methodological choices. However, I will abstain from doing so in this review. For one, it is unlikely that any of my suggestions would supplant the authors’ own perspectives for future studies. On the other hand, within the logic of this manuscript, where that part of the study is manifestly exploratory in nature, any such possible modifications of the methodology might compromise the main goal. All things considered, I found the work reported in this manuscript to be well thought and well balanced, given its two aims (the thoughtful discussion on limitations greatly contributes to this assessment).

Maybe the only section that I found could be slightly improved is the “Materials and methods”. On a first reading, it was not immediately clear to me what parts of the task were similar when assessing the Target and the Friend – only when reading the results did I understand that most questions regarding the Target were also posed for the Friend. If the authors decide to revise this section, it would only improve an already great paper.

In sum, I found this manuscript to be as close to be published in its current from as possible. For that, I congratulate the authors.”

We again thank the reviewer for their positive assessment of our paper. We have revised the manuscript to more clearly communicate that the same questions asked of the target were asked of the friend (see ll. 8).

> “Next, immediately following ratings of the target, participants viewed a photo of a neutral male face (taken from the Radboud Faces Database, Langner et al., 2010) described as a close friend of the previous individual and were asked to estimate the same state and trait emotions (see Figure 1).”
