# Supplementary material for: Possessing potential weapons (still) heightens anger perception: Replicating and extending a test of error management theory
Source: PLoS One. 2025 Oct 17;20(10):e0326446. doi: 10.1371/journal.pone.0326446 (PMC12533867; doi:10.1371/journal.pone.0326446)
Supplement: Attachment — Submitted filename: ReviewResponse.pdf [file pone.0326446.s007.pdf]

Comment to the Editor:

We thank the editor for their comments and feedback on our manuscript following our previous revision.

For data clarity and robustness purposes, the Editor requested us to report several additional measures from the data we obtained in our experiment, including the means and standard deviations of all measures in the study, independent t-tests for state and trait emotion comparisons, and effect sizes for all statistical tests. The Editor also requested that we examine the multivariate normality and homogeneity assumptions for our MANOVA.

We have updated the manuscript to now report these data, as well as the results from the MANOVA assumptions. These are now reported in a new Table 1 (which contains all means and standard deviations for measures in the study), a new Table S1 (which contains results from the independent t-tests between variables and conditions), and columns reporting effect size where appropriate (Tables 2 & 3 and S1). While we found that several assumptions for the MANOVA were not met, the robustness of the MANOVA holds given our relatively large sample size and balanced group sizes, and directly corroborates results from the independent t-tests.

We have also updated our code in the OSF with corresponding changes in this version of the manuscript.

Editor's Comments

1. Currently, the manuscript does not provide a descriptive table summarizing the mean and standard deviation (SD) for all emotion ratings across all conditions (target and friend, armed vs. unarmed conditions). While the OSF script shows that some means and SDs were computed, not all emotion variables appear to be reported. I also noticed that some calculations for descriptive statistics (mean and SD) may be incomplete or inconsistent (https://osf.io/sdaqj, lines 70-90, Date modified: January 29, 2025). I recommend update the script and adding a descriptive statistics table to the results section, ensuring all reported outcome variables are included and clearly reported. For example, structuring a table to include rows for each emotion measure (e.g., state anger, trait anger, state fear, etc.), with separate columns for the experimental conditions (armed/unarmed) and for the target and friend ratings with Mean and SD.

We thank the Editor for their guidance and have added a new Table 1 which shows means and standard deviations for all state and trait emotions along with new text highlighting the table in the main body of the text.

> Lls 159-162:
>
> *As an initial step, we examined descriptive statistics for all emotional and trait ratings by condition (see Table 1), followed by independent samples t-tests to assess differences between the armed and unarmed conditions (see SI Table 1 for full statistical results, including Cohen's d).*

2. Based on the descriptive table suggested in #1, I also recommend reporting the standardized differences and statistical tests (t-tests and Cohen's d) between the armed vs. unarmed conditions. This would provide an initial insight into the experimental effects before the main MANOVA analysis.

We have conducted these analyses, finding convergence with the MANOVA results where raters were more likely to rate the state and trait anger and trait unpleasantness of the target model as higher in the armed condition than in the unarmed condition. We now report these outcomes in a new SI Table 1 and have reported findings from the independent t-tests in the text.

LLs 162-168:

We identified significant differences in state (M_{armed} = 4.36, 162 SD = 2.31; $M_{unarmed}$ = 3.64, SD = 2.00) and trait anger (M_{armed} = 5.36, SD = 1.79; 163 $M_{unarmed}$ = 5.02, SD = 1.61) of the target, as well as in trait unpleasantness (M_{armed} = 4.98, SD = 1.76; $M_{unarmed}$ = 4.31, SD = 1.49). These effects were statistically significant: state anger, $t(457) = 3.64$, $p < .001$, $d = 0.34$; trait anger, $t(460) = 2.19$, $p = .029$, $d = 0.20$; and trait unpleasantness, $t(453) = 4.44$, $p < .001$, $d = 0.41$. All effect sizes fell within the small to approaching moderate range.

3. Before conducting the main analysis, please examine and briefly report the key assumptions of MANOVA, including tests for multivariate normality and homogeneity of covariance matrices.

We have made the recommended additions, including the results of Mardia's test and Box's M. Critically, we found violations of Mardia's test of multivariate normality for the both the target and the friend condition and violations of equality of covariance for the friend condition. Nevertheless, because our data has relatively large sample sizes and equal sized groups and the MANOVA results corroborate those with the independent t-tests, we believe the results MANOVA to be robust, which we discuss in the manuscript.

LLs 169-181:
Prior to conducting the MANOVA analyses, we evaluated whether the assumptions of multivariate normality and homogeneity of covariance matrices were met. Mardia's test indicated significant violations of multivariate normality for both the target and friend rating variables (Target: skewness $\chi^2 = 380.50$, $p < .001$; kurtosis $z = 9.02$, $p < .001$; Friend: skewness $\chi^2 = 725.75$, $p < .001$; kurtosis $z = 13.56$, $p < .001$). However, Box's M test for equality of covariance matrices revealed no significant difference in covariance structures across experimental groups for the target ratings ($p = .141$), suggesting that the assumption of homogeneity was met. In contrast, Box's M test for the friend ratings was significant ($p = .0003$), indicating that the homogeneity of covariance matrices assumption may be violated in that set. While MANOVA is generally robust to moderate departures from multivariate normality and unequal covariances, particularly with balanced group sizes and sufficient sample size, these assumption checks should be considered when interpreting the results.

LLs 191-195:

Importantly, despite assumption violations, the MANOVA results mirrored the pattern observed in the t-tests, with the same three outcome variables—state anger, trait anger, and trait unpleasantness—emerging as statistically significant. This convergence of results across analytical approaches increases confidence in the robustness of the observed effects.

4. For all the MANOVA results (both main text and SOM), please report effect sizes (e.g., eta squared or omega squared) alongside F-values and p-values, and provide corresponding effect size interpretations.

The MANOVA effect sizes are now reported as partial eta squared in the revised text. We have also added descriptions of the effect sizes where appropriate.

LL 168 (with regards to independent t-tests):

All effect sizes fell within the small to approaching moderate range

LL189-191 (with regards to results of the MANOVA):

These effects correspond to small effect sizes, suggesting reliable but modest differences in perceived anger based on the presence of a weapon.

5. In Table 3, the reported values are labeled as β (beta coefficients) and the caption is about regression analysis, but based on the provided description and analysis, these values appear to be correlation coefficients (r). If the analysis was conducted using correlation, the caption should be revised to reflect this. If the authors intend to report regression results (with more than one predictor), run the regression analysis accordingly and report the appropriate regression coefficients separately.

The reporting of the beta coefficients was an error and should instead be Pearson's correlation coefficients. We thought we updated this in the previous version of the manuscript, but those changes were not pushed. We have updated the table to reflect this change.
