# Supplementary material for: Possessing potential weapons (still) heightens anger perception: Replicating and extending a test of error management theory
Source: PLoS One. 2025 Oct 17;20(10):e0326446. doi: 10.1371/journal.pone.0326446 (PMC12533867; doi:10.1371/journal.pone.0326446)
Supplement: Attachment — Submitted filename: Comments to Authors 08122024.docx [file pone.0326446.s006.docx]

Comments to Authors:

The study has two main objectives. Firstly, it aims to replicate Holbrook et al. (2014)’s work and examine whether a target in the armed condition (vs. unarmed condition) is perceived to be angrier. Secondly, it extends prior replication work by exploring whether a friend of the target in the armed condition (vs. unarmed condition) is also appraised as angrier as well.

With the sample size larger than that in Holbrook et al. (2014), the authors replicated the result that participants perceived the target holding a threatening (vs. non-threatening) object as angrier. However, the result was not extended to their friend of the target. Interestingly, the authors found that the affective homophily was present, as the state and trait emotion ratings of both the target and their friend were generally similar.

While I am not an expert in the field of emotion perception, I can understand most parts of the research report. Since this is my first time reading it, I believe the authors could make some slight revisions to enhance clarity, and I have the following suggestions:

#1) In line 78, it would be clearer if the authors replaced the word 'control' with 'unarmed,' assuming that the control condition refers to the unarmed condition. In line 79, the authors can also consider revising the text to clearly state whether the model in the control condition is the same as the model in the unarmed condition.

#2.1) In Figure 1 (Stimuli representing Target and Friend), the authors might consider adding labels for Models 1, 2, and 3 next to each photo of the target’s close friend. While reading the results of the post-hoc Tukey comparisons (lines 160-164), I was unsure which male photo represented Model 3. I assumed the male model with the shortest hair was Model 3.

#2.2) I also assumed the results of post-hoc Tukey comparisons (lines 160-164, and SI Table 1) refer to comparisons of state and trait emotions between friends in the armed condition.

#3) In line 166, the authors wrote ‘We ran multiple linear regressions on friend state and trait anger with both friend state and trait anger and condition as our predictor variables.’.

However, after checking with the data and the syntax, I believe the sentence should be revised to: ‘We ran multiple linear regressions on friend state and trait anger, using both the target’s state and trait anger and condition as our predictor variables.’.
